# Strong Acceptors Based on Derivatives of Benzothiadiazoloimidazole

**DOI:** 10.3390/molecules29102262

**Published:** 2024-05-11

**Authors:** Hanyun Du, Bin Chen, Fengyuan Zhang

**Affiliations:** 1 School of Material Science and Chemical Engineering, Ningbo University, Ningbo 315211, China; duhanyun@nimte.ac.cn; 2 CAS Key Laboratory of Magnetic Materials and Devices, Ningbo Institute of Materials Technology and Engineering, Chinese Academy of Sciences, Ningbo 315201, China; chenbin@nimte.ac.cn

**Keywords:** conjugated compound, naphthalenediimide (NDI), nitrogen heteroacenes, strong acceptor

## Abstract

Despite the rapid progression of organic semiconductors, developing high-air-stability n-type organic semiconductors are still challenging. Herein, novel strong acceptors based on benzothiadiazoloimidazole units are reported. The results reveal that the strong acceptor BTI-NDI-BTI-a has good solubility and high electron affinity (3.94 eV), accompanied by 1D slipped-stacking crystals. Notably, the material presents promising potential for developing into air-stable n-type organic semiconductor materials.

## 1. Introduction

Recently, n-type organic semiconductors have been extensively employed in various domains [1,2,3,4,5] owing to their unique optoelectronic properties and adjustable chemical structures. However, the further development of these devices presents an urgent demand for n-type organic semiconductor materials with air stability and high electron mobility. The stability of n-type semiconductors involves considerations of both thermodynamic and kinetic factors. Thermodynamic analysis underscores the critical importance of a fundamental LUMO energy level (<−3.7 eV) for stability against water, whereas kinetic analysis associated with reduction reactions imposes a higher requirement on the LUMO energy level of the molecular for stability in the presence of oxygen (<−4.9 eV) [6,7,8]. Generally speaking, air-stabilized n-type organic semiconductors maintain their LUMO energy level as low as −4.0 eV to avoid reactions with airborne water and oxygen. Simultaneously, the HOMO energy level is required to be low (<−6.0 eV) to block hole injection [8,9]. Unfortunately, some outstanding n-type organic semiconductors find it difficult to maintain their high mobility in air, such as 4Cl-tetraazapentacenes [10]. Thus, novel design and synthesis principles for air-stable n-type organic semiconductors are strongly required.

There are three primary methods for preparing n-type semiconductors, involving in introducing strong electron-deficient groups, increasing the conjugation length, and introducing heteroatoms into the conjugation skeleton [8,9,11]. Chen et al. reported that thienopyridine (TP) units at naphthalenediimide (NDI) diagonal sites were used to obtain a new electron-deficient unit, thienopyridine-fused naphthalene amide (TPNA), with low HOMO energy levels (−5.8 eV~−6.0 eV), but the LUMO energy levels were still high (−3.27 eV~−3.38 eV) [12]. Jenekhe et al. reported phenazine-substituted poly(benzimidazo-benzophenanthroline) (BBL-P) with a low LUMO energy level of −4.0 eV, but its HOMO energy level was −5.5 eV, as derived by cyclic voltammetry [13]. These results indicate that increasing the conjugation length and introducing heteroatoms can either lower the LUMO energy level of the conjugated system while raising the HOMO energy level or lower the HOMO energy level while raising the LUMO energy level [12,13,14]. Therefore, a new strategic approach is required to simultaneously lower the LUMO/HOMO energy levels in order to enhance the performance of n-type organic semiconductors.

**Scheme 1:**
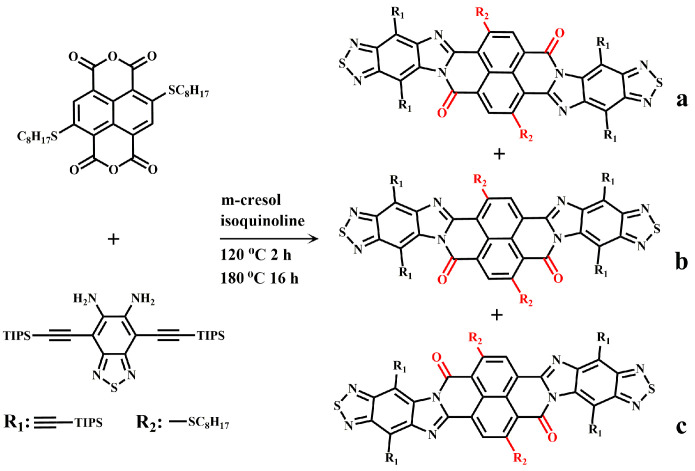
Synthesis of BTI-NDI-BTI (**a**–**c**).

Herein, a novel approach exploring the combination of low LUMO/HOMO energy levels with enhanced molecular packing was investigated. The extensively researched electron-deficient units benzothiadiazole (BT) [15,16] and NDI [17,18] were successfully integrated to obtain the novel acceptor unit BTI-NDI-BTI. The BTI-NDI-BTI units have lower LUMO/HOMO energy levels. The BTI-NDI-BTI units form a tightly packed structure through intermolecular noncovalent bonds (N···N/N···S) to ensure their high mobility. To tune the solubility and crystallinity of the molecules, the octylthio and triisopropylsilyl (TIPS) acetylenic groups are introduced into the NDI core and BT sites, respectively. The introduction of side groups theoretically leads to the appearance of three regionally isomeric molecules (a, b, and c), as shown in Scheme 1. In the end, only the pure molecular BTI-NDI-BTI-a unit is successfully isolated, exhibiting lower HOMO/LUMO energy levels. Meanwhile, single-crystal X-ray diffraction reveals the 1D slipped-stacking.

## 2. Results and Discussion

### 2.1. The Synthesis of BTI-NDI-BTI

**Scheme 2:**
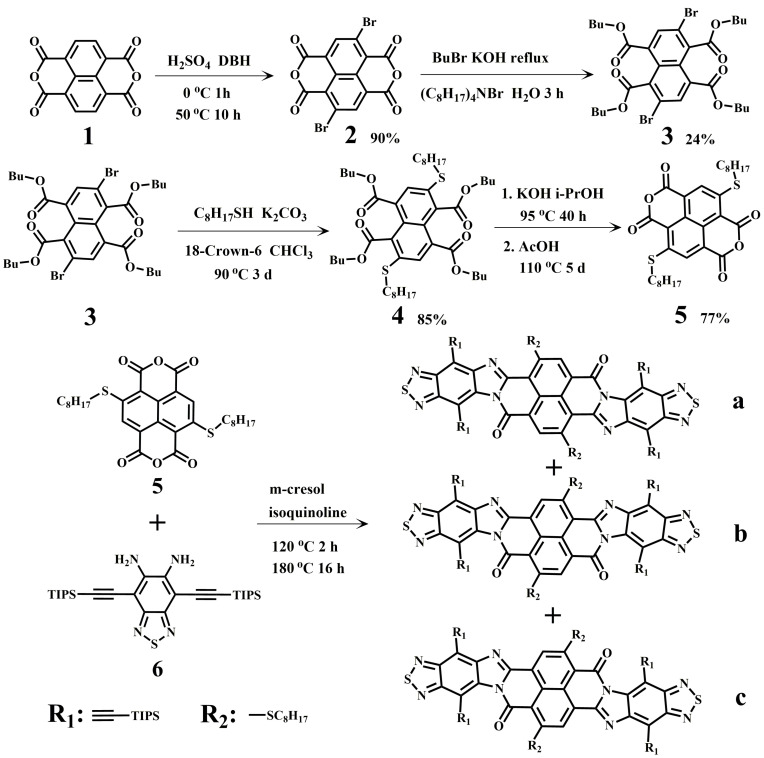
Synthesis process for BTI-NDI-BTI.

The full synthesis process of BTI-NDI-BTI is illustrated in Scheme 2. The precursors of compounds were synthesized based on detailed procedures in the previous literature [19,20]. As demonstrated in Scheme 1, the condensation reaction of compounds could theoretically be used to obtain three isomers of BTI-NDI-BTI. These new structures were characterized by high-resolution mass spectrometry (HRMS) and NMR spectroscopy (Figures S1–S6). In the ^1^H NMR spectrum of BTI-NDI-BTI (Figure S3), peaks corresponding to C-H (8.91 ppm) and S-CH_2_ (3.33–3.31 ppm) were clearly observed. In the ^13^C NMR spectrum of BTI-NDI-BTI (Figure S4; the peaks facing down represent primary carbons and tertiary carbons), peaks corresponding to C-H (128.70 ppm) in the bay position and CH_3_-CH-CH_3_ (19.12, 18.98, 11.79, and 11.66 ppm) on TIPS were also clearly observed, which verified the successful synthesis of BTI-NDI-BTI. The HRMS also verified the successful synthesis of BTI-NDI-BTI. As Figure S7 illustrates, the measured mass-to-charge ratio (*m*/*z*) of the molecular ion peak was 1537.7582, accompanied by the highest signal intensity. The error between the measured *m*/*z* value and the fitted result was a mere 0.001, falling within the acceptable error threshold for HRMS. Therefore, combining the NMR results, it is unequivocally established that BTI-NDI-BTI was prepared successfully. However, the results did not provide clarification regarding whether it was BTI-NDI-BTI-a or BTI-NDI-BTI-c.

### 2.2. The Results of Crystals

**Figure 1:**
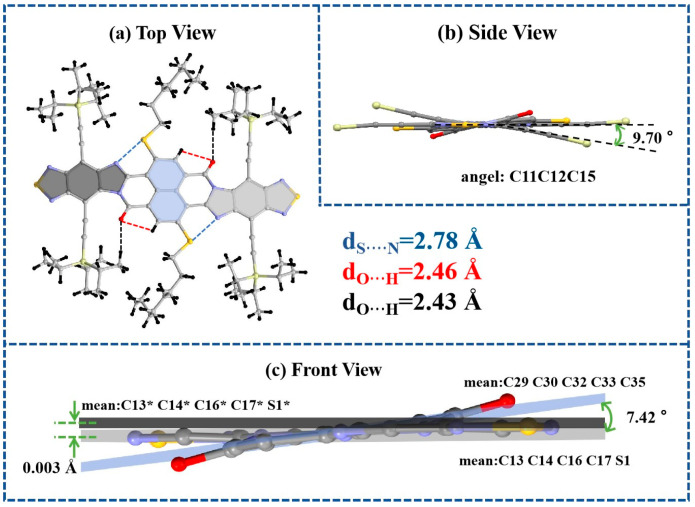
Crystalline configuration of BTI-NDI-BTI-a. (**a**) top view; (**b**) side view; and (**c**) front view. The red-, yellow-, grey-, black-, blue-, and light-yellow-colored atoms represent O, S, C, H, N, and Si, respectively. And * is used to distinguish carbon atoms on symmetric structures.

Fortunately, red crystals were acquired via the slow evaporation of tetrahydrofuran at room temperature. The results exhibited that the obtained molecule was BTI-NDI-BTI-a, as demonstrated in Table S1. In a single molecule, interactions between N···S and H···O are observed (Figure 1a). As shown in Figure 1b, a noticeable bending of the TIPS acetylenic group is observed on the side corresponding to the carbonyl group. This is attributed to the steric hindrance effect of the TIPS acetylenic group, which disrupts the planarity of the conjugated backbone. Meanwhile, two benzothiadiazoles are parallel, and their planar spacing is 0.003 Å. And these two parallel structures intersect at the naphthalene ring plane. The dihedral angle is 7.42° at the intersection (Figure 1c).

**Figure 2:**
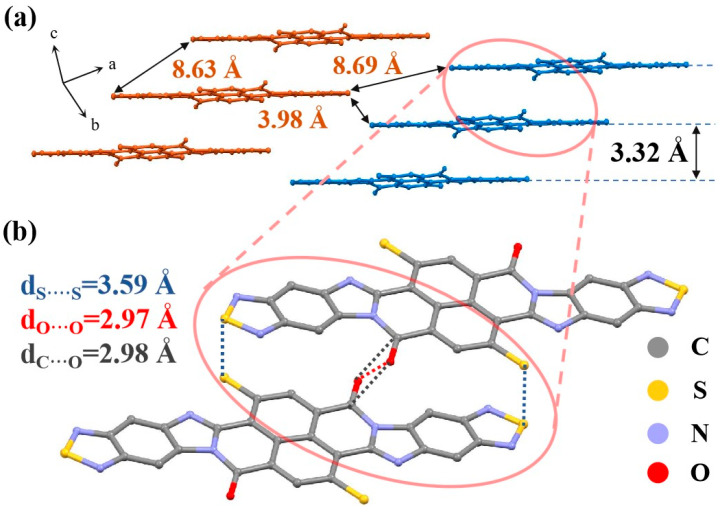
(**a**) One-dimensional slipped stacking of BTI-NDI-BTI-a crystals (TIPS-acetylene and octane units were removed for clarity); (**b**) different noncovalent bond intermolecular interactions.

The main skeletons of BTI-NDI-BTI-a are found by 1D slipped-packing with a π–π stacking distance of 3.32 Å in Figure 2a. Specific short contacts are observed between the stacks, including S···S (3.59 Å), O···O (2.97 Å), and O···C (2.98 Å), as shown in Figure 2b [21,22]. However, no π–π packing is observed along the other two axes (Figure S7), which is attributed to the substantial repulsion between the TIPS groups.

### 2.3. The Results of UV/Vis Absorption Spectrum and Cyclic Voltammetry

**Figure 3:**
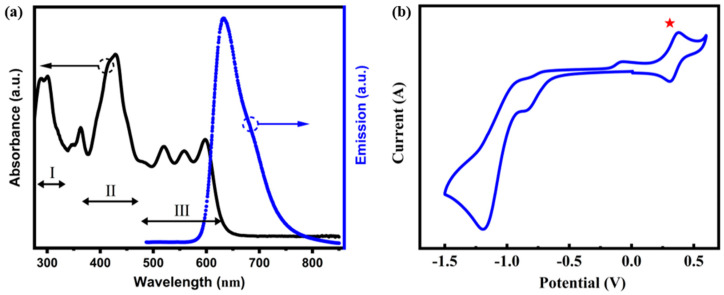
(**a**) UV/Vis absorption and PL spectrum of BTI-NDI-BTI-a in DCM. (**b**) Cyclic voltammograms of BTI-NDI-BTI-a in DCM, with ferrocene (the ferrocene peak is indicated by a red star and occurs at a positive potential) as an internal standard.

BTI-NDI-BTI-a was diluted in dichloromethane (DCM) to investigate its UV/Vis absorption spectrum. As demonstrated in Figure 3a, three main absorption bands are observed, which are at 270 to 350 nm (Band Ⅰ), 370 to 460 nm (Band Ⅱ), and 490 to 640 nm (Band Ⅲ), respectively. The absorption profiles of the molecule are similar to the β, p, and α bands of polycyclic hydrocarbons, as described by Clar [23]. Thiadiazoloquinoxaline derivatives also exhibit multiple peaks which are similar to those in Band Ⅲ [20]. Bands Ⅰ and Ⅱ are attributed to the π–π* and n–π* transitions of the conjugated aromatic segments [24]. In addition, Band Ⅲ corresponds to intramolecular charge transfer. The onset of the absorption of BTI-NDI-BTI-a is at 636 nm, and the corresponding optical gap is deduced to be 1.95 eV, as demonstrated in Table 1. In addition, the normalized photoluminescence (PL) spectrum of BTI-NDI-BTI-a in DCM is also presented in Figure 3a. Upon excitation at a wavelength of 467 nm, BTI-NDI-BTI-a exhibits a red fluorescence, and its fluorescence quantum yield (ϕf) is 5.27%. The peak emission wavelength is at 632 nm.

The cyclic voltammetry curve is demonstrated in Figure 3b. Two reduction peaks are observed in the negative direction. The first onset reduction potential of BTI-NDI-BTI-a is determined to be −0.63 V. The corresponding electron affinity (EA) is calculated to be 3.94 eV using the equation provided in the supporting information. The small signal around −0.05 is caused by external impurities.

Furthermore, the electronic structure of BTI-NDI-BTI-a was investigated by density functional theory (DFT) calculations and time-dependent DFT (TD-DFT). As shown in Figure S10, the molecular geometry and LUMO/HOMO levels of the BTI-NDI-BTI derivatives are calculated at the DFT-B3LYP/6-31G(d) level. The LUMO/HOMO levels are −3.57 eV and −5.58 eV. Excited-state transitions were calculated using TDDFT at the B3LYP/6-31G(d) level of theory. The calculated absorption spectrum is displayed in Figure S11. This closely resembles the experimental absorption spectrum, where the low-energy peak centered at 377 nm represents the π–π* transition. Pictorial representations of both the electron and hole orbital distributions associated with the π–π* transition are shown in Figure S12. These results are consistent with experimental values. In addition, the low electron affinity of BTI-NDI-BTI-a demonstrates its potential for constructing air-stable n-type organic semiconductor molecules.

## 3. Materials and Methods

### 3.1. Materials

The full synthesis process of BTI-NDI-BTI is illustrated in Scheme 2. Solvents were purified with the usual procedure before use. The other materials are common commercial-level materials and were used as received. 2,6-dibromo-1,4,5,8-naphtalenetetracarboxylic dianhydride (**2**) [25], 2,6-dibromo-1,4,5,8-tetra(n-butoxycarbonyl)naphthalene (**3**) [26], 2,6-bis(octylthio)naphthalene-1,4,5,8-dianhydride (**5**) [19], and 5,6-diamine-4,7-bis((triisopropylsilyl)ethynyl)-benzo[c][1,2,5]-thiadiazole (**6**) [20] were prepared according to the reported procedures.

Synthesis of 2,6-bis(octylthio)naphthalene-1,4,5,8-tetracarboxylic tetrabutyl-ester (4)

The synthesis of **4** was modeled after a procedure in the literature [19]. 2,6-dibromo-1,4,5,8-tetra (n-butoxycarbonyl) naphthalene (3, 686.43 mg, 1 mmol, 1 eq), K_2_CO_3_ (2.78 g, 20 mmol, 20 eq), 1-octanethiol (8.67 mL, 7.31 g, 50 mmol, 50 eq), and a catalytic amount of 18-crown-6 (27.6 mg, 105 μmol, 0.1 eq) were suspended in anhydrous CHCl_3_ (18 mL) in a pressure-resistant flask. The flask was sealed and heated to 90 °C for 3 days. After cooling to room temperature, the mixture was treated with sat. aq. NaHCO_3_ (40 mL). The organic layer was separated, and the aqueous layer was extracted with CHCl_3_ (3 × 40 mL). The combined organic layers were dried over MgSO_4_, filtered, and concentrated in vacuo. The crude yellow product was purified by column chromatography (silica gel, petroleum ether/DCM 1:3) to yield compound **4** (695.12 mg, yield 85%) as a bright yellow solid. Mp = 63.6–65.0 °C. ^1^HNMR (CDCl_3_, 600 MHz) δ: 7.87 (s, 2H), 4.30–4.27 (m, 8H), 3.00–2.98 (t, 4H, 7.5 Hz), 1.75–1.71 (m, 8H), 1.65–1.63 (m, 4H), 1.46–1.42 (m, 8H), 1.28–1.25 (m, 20H), 0.97–0.94 (m, 12H), and 0.88–0.85 (m, 6H). ^13^CNMR (CDCl_3_, 600 MHz) δ: 167.8, 166.5, 137.4, 131.8, 131.7, 129.4, 127.2, 65.7, 65.6, 33.9, 31.9, 30.6, 30.5, 29.3, 29.2, 29.1, 29.0, 22.7, 19.4, 19.3, 14.2, and 13.8. HRMS (APCI): the *m/z* for C_46_H_73_O_8_S_2_[M+H^+^] 817.4747 was found to be 817.4655.

Synthesis of BTI-NDI-BTI-a

Under an argon atmosphere, a solution of 2,6-bis (octylthio) naphthalene-1,4,5,8-tetracarboxylic dianhydride (**5**, 38.4 mg, 0.1 mmol), 5,6-diamine-4,7-bis ((triisopropylsilyl) ethynyl) benzo-[c][1,2,5]thiadiazole (**6**, 108 mg, 0.23 mmol), and isoquinoline (0.15 mL) in m-cresol (1 mL) were heated at 120 °C for 2 h and then heated up to 180 °C for 16 h. After cooling to room temperature, the solvents were removed in a vacuum, and the residue was purified by silica gel chromatography with petroleum ether/dichloromethane (1:1, R_f_ = 0.38) as an eluent. There were similar R_f_ values between BTI-NDI-BTI-b and BTI-NDI-BTI-c, as shown in Figure S8. In addition, there were insoluble, one-sided products. So, the pure BTI-NDI-BTI-a was obtained as a red solid (6.3 mg, yield 4%). Additionally, BTI-NDI-BTI-a had good solubility in common organic solvents (>5 mg/mL in DCM, CHCl_3_, and THF), as shown in Figure S9. The excess integrals in the adipose zone spectra were caused by the residual petroleum ether. Mp > 300 °C. ^1^HNMR (CDCl_3_, 600 MHz) δ: 8.91 (s, 2H), 3.33–3.30 (t, 4H, *J* = 7.86 Hz), 1.97–1.95 (m, 4H), 1.66–1.63 (m, 4H), 1.44–1.42 (m, 4H), 1.35–1.25 (m, 92H), and 0.89–0.84 (m, 6H). ^13^CNMR (CDCl_3_, 600 MHz) δ: 157.32, 155.68, 153.81, 153.06, 149.99, 146.17, 134.12, 128.70, 125.50, 124.19, 118.61, 110.55, 107.63, 107.28, 103.51, 100.21, 99.87, 33.20, 32.01, 29.86, 29.67, 29.50, 29.36, 28.38, 22.85, 19.12, 18.98, 14.24, 11.79, and 11.66. HRMS (APCI): the *m*/*z* for C_86_H_121_N_8_O_2_S_4_Si_4_[M+H^+^] 1537.7572 was found to be 1537.7582.

### 3.2. Methods

^1^HNMR and ^13^CNMR (attached proton test, APT) spectra were recorded in deuterated solvents with a Bruker (Billerica, MA, USA) AVANCE NEO 600 Spectrometer. High-resolution mass spectrometry (HRMS) was performed via atmospheric pressure chemical ionization (APCI). Single-crystal X-ray crystallographic data for the molecules were collected with a Bruker D8 VENTURE dual-wavelength Ga diffractometer. The crystal was kept at 193.00 K during data collection.

UV-Vis absorption spectra were measured with a Lambda 950 spectrophotometer (PerkinElmer, Waltham, MA, USA) at room temperature. The cyclic voltammetry (CV) curve was plotted using an electrochemical analyzer with a three-electrode cell (Shanghai Chenhua Instrument Co., Ltd., Shanghai, China, CHI600E) in an anhydrous dichloromethane solution of n-Bu_4_NPF_6_ (0.1 M) with a scan rate of 10 mV/s at room temperature under an argon atmosphere. A glassy carbon electrode was used as the working electrode, while platinum wires were used as a counter electrode and a reference electrode, respectively. The voltammogram was recorded and referenced to the ferrocene/ferrocenium (Fc) redox couple as an internal standard.

Density functional theory (DFT) calculations were performed using the Gaussian 16 program [27] with the B3LYP hybrid functional [28] and basis set 6-31G(d) for the ground-state geometry optimization. TDDFT calculations were performed using the Gaussian 16 program with the B3LYP hybrid functional and basis set 6-31G(d) for the ground-state geometry optimization. Both isopropyl and octyl groups in the molecule were replaced by methyl groups for simplification.

The optical gap and electron affinity were calculated according to the following equations:

(1) $$E_{gap}^{opt}=1240/\lambda_{abs}$$

*EA* = 4.8 + (*E*_onset_ − *E*_Fc_) (2)

## 4. Conclusions

In summary, a novel electron-deficient acceptor (BTI-NDI-BTI) was successfully synthesized. More importantly, its derivative (BTI-NDI-BTI-a) was successfully synthesized and isolated. BTI-NDI-BTI-a displays good solubility. The experimental results indicate that it has high electron affinity (3.94 eV) without strong withdrawing groups like fluorine and cyano groups. And the presence of large conjugation planes with S···S/S···N noncovalent interactions facilitates 1D slipped-stacking. In comparison with other compounds [12,13,29], all these findings suggest that BTI-NDI-BTI has promising potential for air-stable n-type organic semiconductors. Further investigation is ongoing in our laboratory. Last but not least, this study provides a novel strategy for fabricating n-type organic semiconductors with low molecular energy levels.

## Figures and Tables

**Table 1:** Photophysical and electrochemical properties and energy levels of BTI-NDI-BTI-a.

	$E_{red}^{onset}\left(V\right)$ ^a^	$E_{g}^{opt}$(eV) ^b^	EA (eV) ^c^	E_LUMO_ (eV) ^d^	E_HOMO_ (eV) ^d^
BTI-NDI-BTI-a	−0.63	1.95	3.94	−3.57	−5.58

^a^ Measured in an *n*-Bu_4_NPF_6_ solution in dichloromethane with a scan rate of 10 mV/s and ferrocene as an internal standard. ^b^ Estimated from absorption onset. ^c^ The energy of Fc/Fc+ was assumed to be −4.8 eV relative to a vacuum. ^d^ Calculated energy levels using B3LYP/6-31G(d).

## Data Availability

Data are contained within the article.

## Supplementary Materials

The following supporting information can be downloaded at https://www.mdpi.com/article/10.3390/molecules29102262/s1: NMR spectra (Figures S1–S4), HRMS analyses (Figures S5–S6), R_f_ values (Figure S8), solubility (Figure S9), DFT results (Figures S10–S12), and crystal data (Table S1 and Figure S7). Refs. [30,31,32] are cited in Supplementary Materials.
