# Supplementary material for: Strong Acceptors Based on Derivatives of Benzothiadiazoloimidazole
Source: Molecules. 2024 May 11;29(10):2262. doi: 10.3390/molecules29102262 (PMC11124087; doi:10.3390/molecules29102262)
Supplement: Supplementary file 1 [file molecules-29-02262-s001.zip › molecules-2994423-supplementary.pdf]

Supporting Information

Strong Acceptors Based on Derivatives of Benzothiadiazoloimidazole

Hanyun Du [1], Bin Chen [2] and Fengyuan Zhang [2,*]

[1] School of Material Science and Chemical Engineering, Ningbo University, Ningbo 315211, China; duhanyun@nimte.ac.cn

[2] CAS Key Laboratory of Magnetic Materials and Devices, Ningbo Institute of Materials Technology and Engineering, Chinese Academy of Sciences, Ningbo 315201, China; chenbin@nimte.ac.cn

* Correspondence: zhangfengyuan@nimte.ac.cn

Contents

1. Characterization Data........1

1.1 NMR spectra........1

1.2 HRMS spectra........3

2. Crystal data........4

3. R_f values and Solubility........6

4. DFT and TDDFT results........7

5. References........9

1. Characterization Data

1.1 NMR spectra

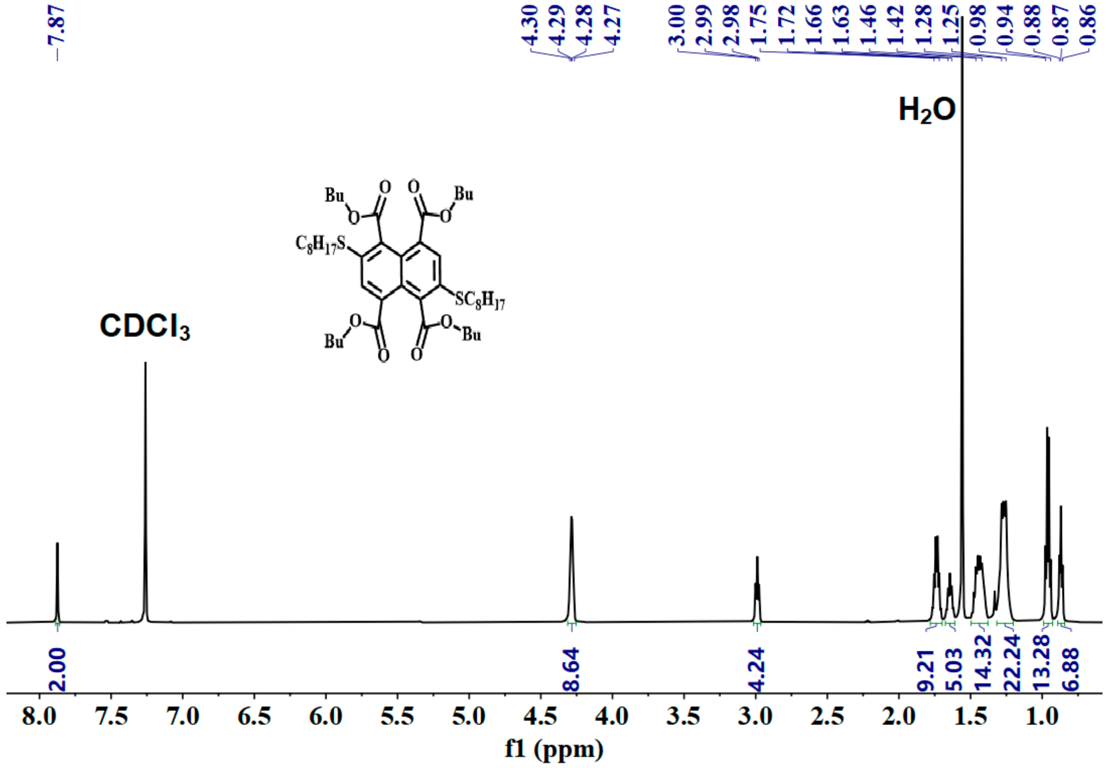

Figure S1. ^{1}HNMR (600 MHz, $CDCl_3$) spectrum of **compound 4**.

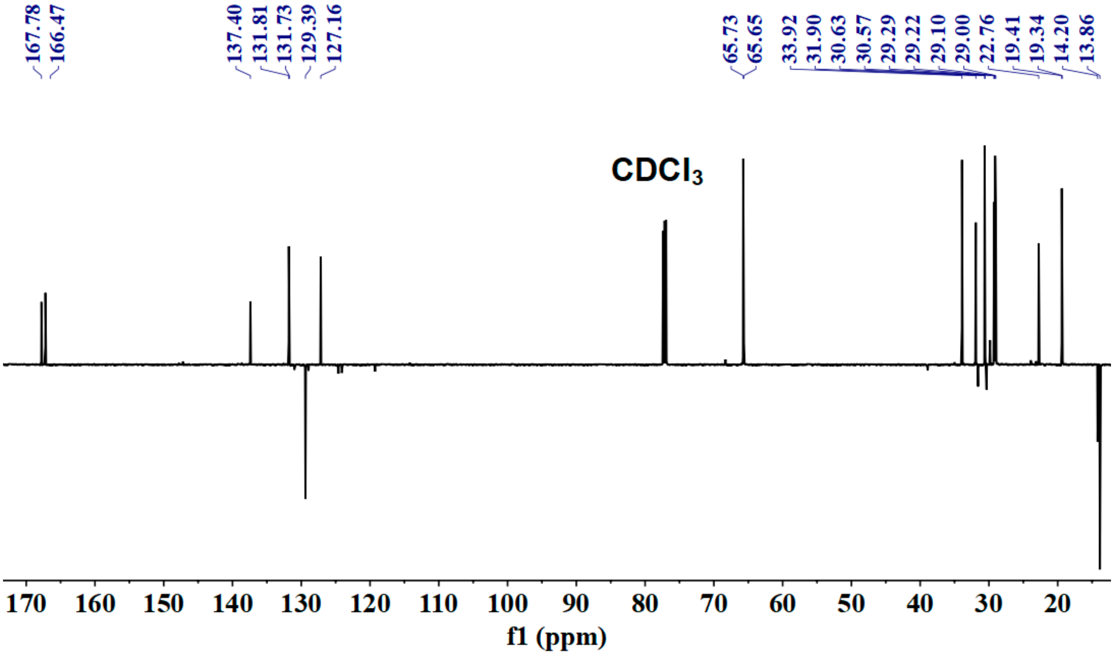

Figure S2. The APT ^{13}CNMR (600 MHz, $CDCl_3$) spectrum of **compound 4**.

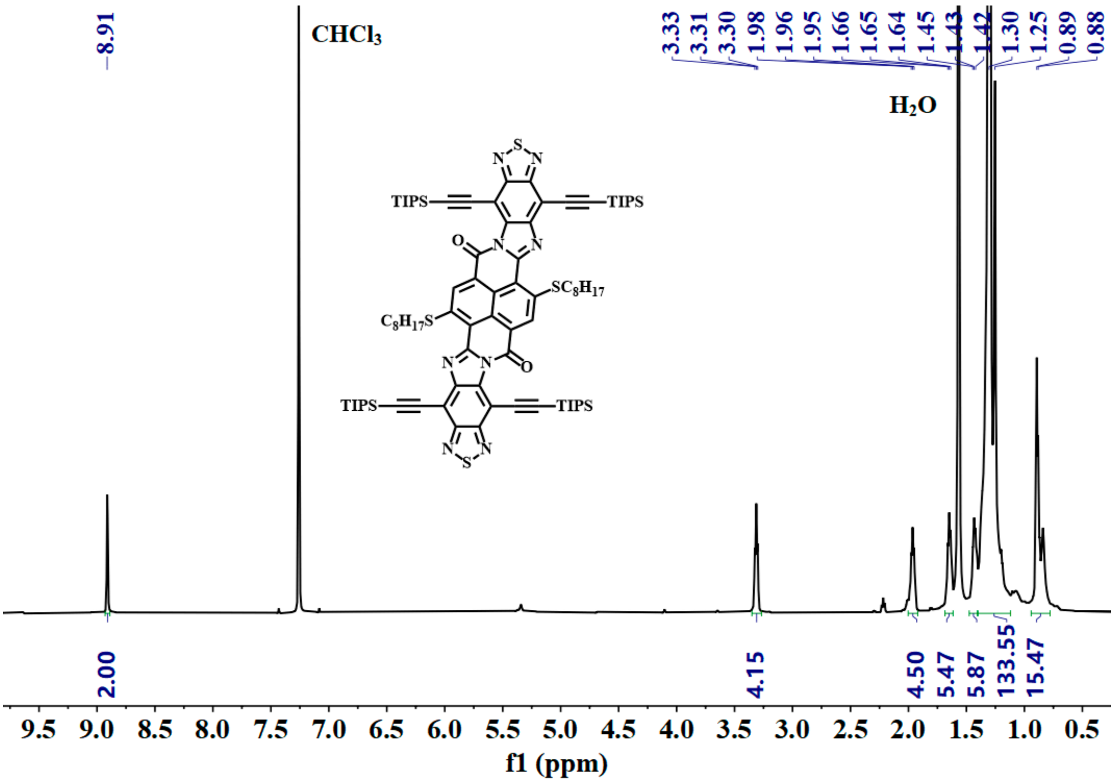

Figure S3. ^{1}HNMR (600 MHz, $CDCl_3$) spectrum of **BTI-NDI-BTI-a**.

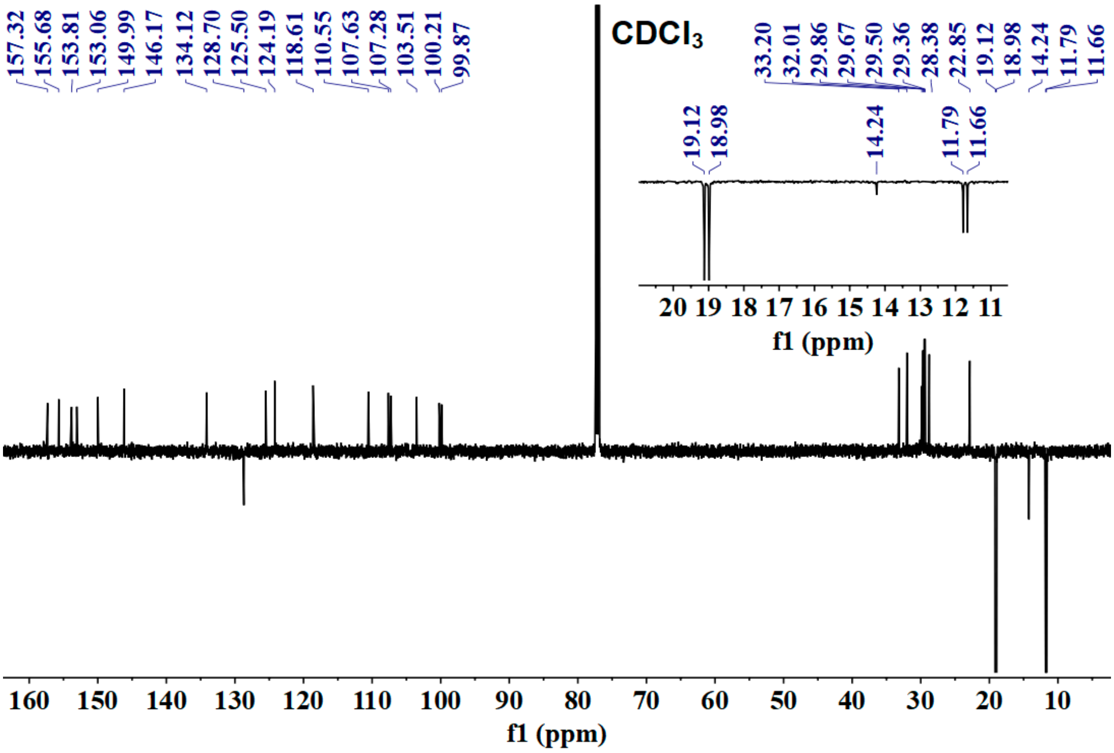

Figure S4. The APT ^{13}CNMR (600 MHz, $CDCl_3$) spectrum of **BTI-NDI-BTI-a**.

1.2 HRMS spectra

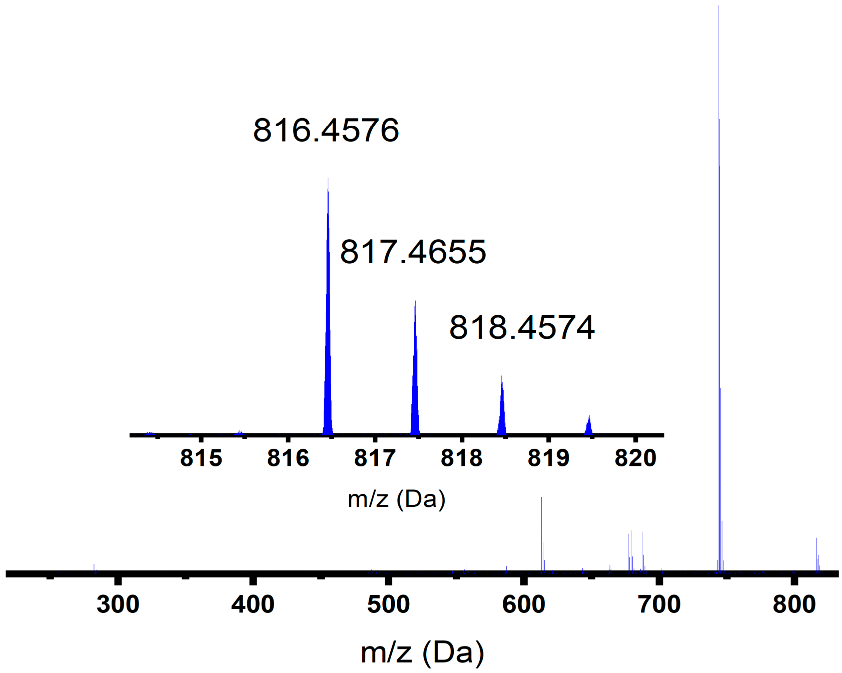

Figure S5. HRMS of **compound 4**.

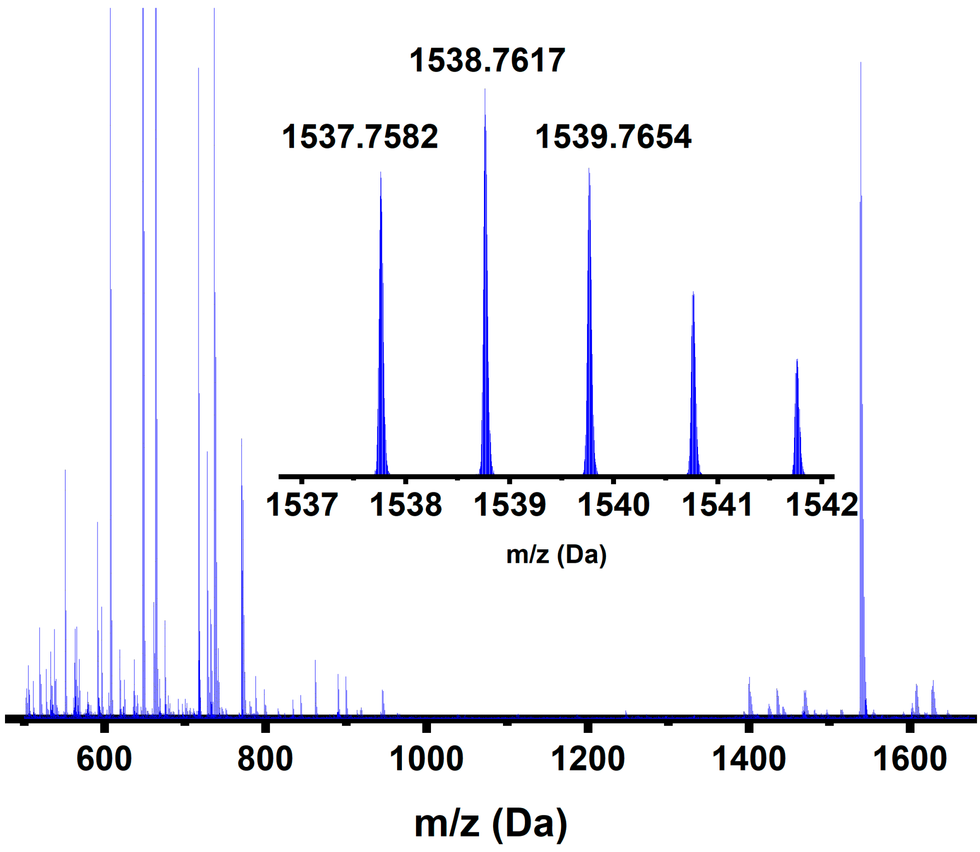

Figure S6. HRMS of **compound BTI-NDI-BTI-a**.

2. Crystal data

Table S1. Crystal data of **BTI-NDI-BTI-a**

Compound	BTI-NDI-BTI-a
CCDC number	2321766
Chemical formula	$C_{80}H_{120}N_8O_2S_4Si_4$
Crystal system	triclinic
Space group	$P\bar{1}$
No. of reflections measured	15256
a (Å)	8.6351(11)
b (Å)	14.9947(18)
c (Å)	17.843(2)
α (°)	106.688(8)
β (°)	91.582(8)
γ (°)	99.525(8)
Z	2
Density (g cm^{-3})	1.174
Unit cell volume / $Å^3$	2175.6(5)
Temperature (K)	193.00
R1 [I>=2σ(I)]	0.0818
wR2	0.2760
R (reflections)	0.0852

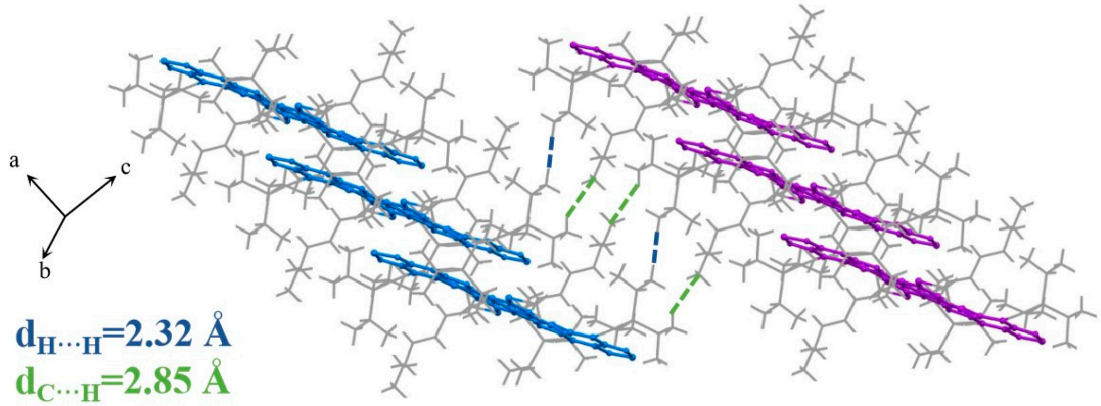

Figure S7. Aggregation pattern of **BTI-NDI-BTI-a**. Grey chain segments indicate thioether side groups and TIPS acetylene groups.

3. R_f values and Solubility

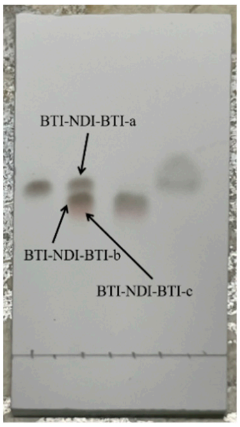

Figure S8. R_f values of obtained products.

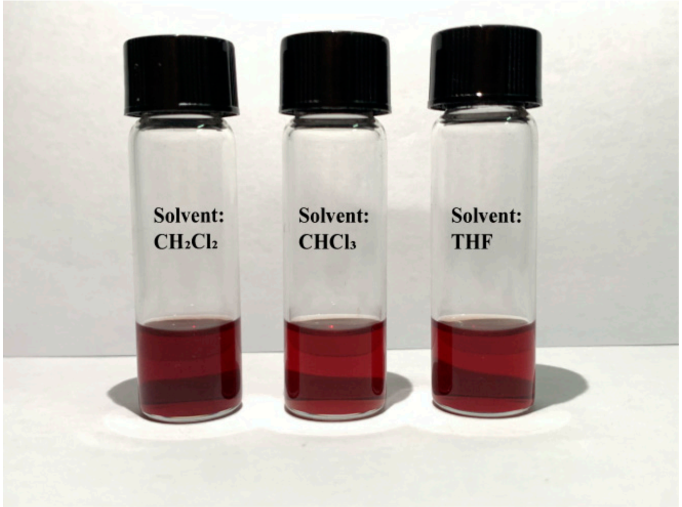

Figure S9. The solubilities of **BTI-NDI-BTI-a** in different solvents (CH_2Cl_2, $CHCl_3$, and THF in 1 ml bottle).

4. DFT and TDDFT results

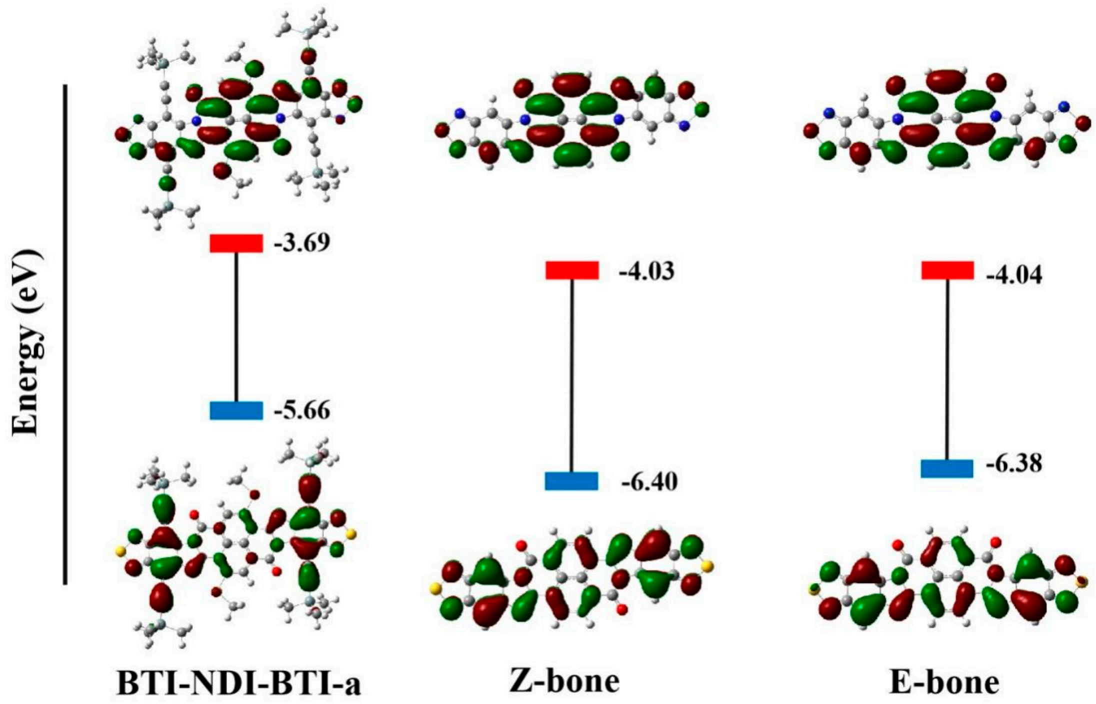

Figure S10. Molecular geometry and LUMO/HOMO levels of **BTI-NDI-BTI** derivatives calculated at the DFT-B3LYP/6-31G level on Multiwfn program[1].

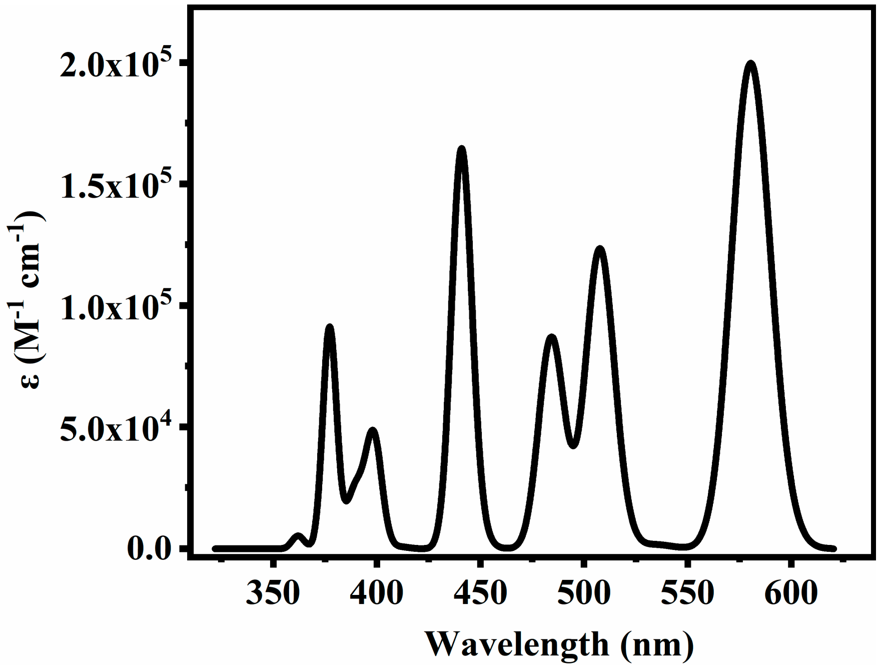

Figure S11. Simulated absorption spectrum of the gas phase **BTI-NDI-BTI-a** calculated at the time-dependent (TD) B3LYP/6-31G(d) level of theory calculated with 20 excited states.

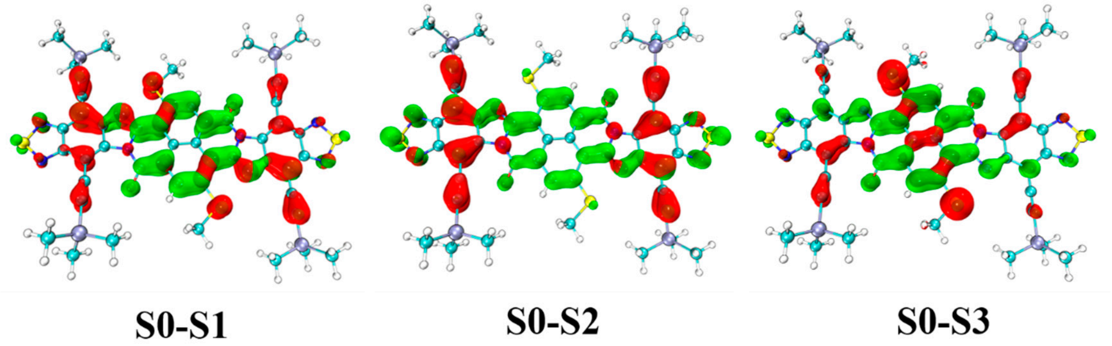

Figure S12. Distribution of electrons and holes in different excited states [2]. (red for holes, green for electrons, graphing with VMD [3])

5. References

1. Lu, T.; Chen, F.W. Multiwfn: A multifunctional wavefunction analyzer. *J. Comput. Chem.* **2012**, *33*, 580-592, doi:10.1002/jcc.22885.
2. Liu, Z.Y.; Lu, T.; Chen, Q.X. An sp-hybridized all-carboatomic ring, cyclo[18]carbon: Electronic structure, electronic spectrum, and optical nonlinearity. *Carbon* **2020**, *165*, 461-467, doi:10.1016/j.carbon.2020.05.023.
3. Humphrey, W.; Dalke, A.; Schulten, K. VMD: Visual molecular dynamics. *J Mol Graph Model* **1996**, *14*, 33-38, doi: 10.1016/0263-7855(96)00018-5.
